# Editorial: Integrating Predation Risk Across Scales: from Neurons to Ecosystems and Milliseconds to Generations

**DOI:** 10.3389/fnbeh.2020.00042

**Published:** 2020-03-31

**Authors:** Jacqueline J. Blundell, Evan L. Preisser

**Affiliations:** ^1^Department of Psychology, Memorial University of Newfoundland, St. John's, NL, Canada; ^2^Department of Biological Sciences, University of Rhode Island, Kingston, RI, United States

**Keywords:** predation risk, predator, prey, defense, fear

This editorial review of the Research Topic “Integrating predation risk across scales: from neurons to ecosystems and milliseconds to generations” explores prey responses to predation risk from an array of different perspectives. It includes work from neurobiologists, ecologists, and other disciplines interested in predator-prey interactions at varying spatial and temporal scales. Taken together, the 15 papers in this Research Topic represent an attempt to synthesize work across disciplines in search of intellectual synergies and new avenues of research collaboration.

## Ecological Responses To Predation Risk: Original Research

Papers by Madin et al. and Atwood et al. report on research addressing the phenomena of coral reef “halos,” zones of unvegetated seafloor surrounding coral reef patches. These patches have long been thought to result from herbivorous fish seeking shelter from predators within reef cracks and crevices; they only feed on vegetation growing near the coral because foraging too far from shelter might expose them to predators. Madin et al. report that coral reef halos found in the Great Barrier Reef result from both prey fear and the action of bioturbators, sediment-dwelling organisms that churn up the seafloor and affect algal settlement. Atwood et al. conducted complementary research confirming the importance of predation risk in changing algal grazing rates and demonstrating that these changes decrease sedimentary carbon storage within the reef halos.

Both Chizzola et al. and Bleicher et al. describe work exploring the “landscape of fear” in terrestrial systems. Fear of lions has long been thought to shape the habitat choices and foraging decisions of many herbivorous mammals; Chizzola et al. combine behavioral, physiological, and isotopic information to reveal the highly dynamic and scale-dependent responses of wildebeest and impala to this fearsome threat. At a smaller spatial scale, Bleicher et al. explore how desert rodents on two continents respond to the common threat of owls and snakes, two dangerous but very different predators. They found that the two rodent communities differ in their assessment of which predator is the most threatening and discuss how local environments can profoundly shape prey responses.

The special feature also contains several articles exploring the impact of risk on individuals. First, Trakimas et al. discuss the connections between development rate and physiological stress in the cricket *Gryllus integer*. They found that slow-developing crickets had higher resting metabolic rates than fast-developing ones, presumably reflecting the energetic costs associated with a higher resting metabolism. They also resumed activity faster following a stressor than did fast developers, a relationship that may translate into higher foraging rates. Second, Baranowski and Preisser report the results of work testing the impact of predation risk on the survival of luna moth (*Actias luna*) larvae. They found that caterpillars exposed to a threatening but non-lethal predator (a wasp with its jaws and stinger immobilized) would “freeze” in place, stop feeding, and die at a higher rate than larvae exposed to a similarly-sized but harmless fly. Finally, Eason et al. describe fascinating work addressing how threat type and refuge availability affect the trajectory of fleeing Eastern Gray Squirrels (*Sciurus carolinensis*). Briefly, they found that squirrels preferred not to run to refuges (trees) that were directly behind them and would actually run toward threats to reach a tree in front of them.

## Ecological Responses To Predation Risk: Literature Reviews

The Research Topic includes two large-scale reviews of different ecological processes that affect predator-prey interactions. In the first, Weiss summarizes our current understanding of how *Daphnia*, a crustacean that is a model system for both ecology and evolutionary biology, detect and avoid predators. In addition to defending themselves against predators, Daphnia exposed to predator cues can produce offspring with spines and “helmets” that make them more difficult for predators to subdue and consume. The review traces response from the initial cues to the underlying neurophysiological mechanisms that structure prey responses, providing a concise overview of the molecular mechanisms underlying both short- and long-term phenotypic adaptation. In the second, Draper and Weissburg provide a review and synthesis of the impact of global warming and increased CO_2_ levels on the sensory ecology of predator-prey interactions. Both factors affect multiple parts of the predator-prey encounter sequence, including the ability of predators to detect prey and the ability of prey to flee approaching predators. Although cue production, transmission, and reception will be globally affected by such large-scale environmental changes, they highlight the fact that aquatic predator-prey interactions may be disproportionately sensitive to these disruptions. Altered CO_2_ levels are likely to have especially strong effects in marine systems due to ocean acidification, which can change Ph levels sufficiently to make the calcium-based shells of prey difficult or impossible to produce.

## Neurobiological Responses To Predation Risk: Original Research

Moths and other night-flying prey are often targeted by bats that use ultrasonic calls to detect and help capture them. As a result, many moths possess tympanic organs capable of detecting bat sonar.  Cinel and Taylor explored how chronic exposure to bat calls affected transcription in fall armyworms (*Spodoptera exigua*) that were either continuously exposed to foraging and attack calls or held in silence. The 290 transcripts they found either up- or down-regulated were involved in a broad range of cellular functions, information that lays the foundation for research addressing how these changes affect insect physiology, behavior, and demography. Working in a very different system, Tong et al. report on how testosterone affects both mouse fear and physiology. Exposing male mice to this chemical reduced their innate fear of predator cues (cat urine) when allowed to explore potentially risky habitats. In a complementary experiment, they also found that testosterone produced hypomethylation of the promoter region of the arginine vasopressin gene. The latter compound plays a key role in socio-sexual behavior; they suggest that testosterone-induced reductions in fear occur via manipulations of the trade-off between fear (reduced activity) and social/sexual interactions (increased activity).

## Neurobiological Responses To Predation Risk: Literature Reviews

Recent technical advances in neurobiology have revolutionized our understanding of how predators and prey detect and respond to each other. This progress has heightened our appreciation for the role of the periaqueductal gray (PAG) in modulating such interactions; Franklin provides a cogent assessment of our current understanding. Work in rodent model systems demonstrates the role of the rostrolateral PAG in hunting behaviors and how the dorsal-ventral and rostral-caudal axes of the PAG differ in their impact on defensive behavior. The fact that brain imagining work in humans shows threat-induced PAG activity suggests that large portions of PAG function are likely conserved across an array of mammalian species.

The amygdala, and the basolateral neurons within it, also play a critical role in detecting and responding to predator cues; Mitra reviews the role of these neurons and how they change in response to predator odors. While neurons in the basolateral amygdala respond quickly to risk cues, continued exposure to stress-related hormones can change them in ways that also facilitate rapid endocrine responses to future stressors. Current evidence suggests that the basolateral amygdala plays an important role in risk-related activities across multiple taxa, and that it may be generally responsible for anticipatory defensive behaviors in threatening situations.

Fear generalization is an adaptive process that promotes survival in complex and dynamic environments. In their review, Asok et al. explore the behavioral, neural, genetic, and biochemical mechanisms involved in this process. As fear generalization is a hallmark of many anxiety and stress-related disorders (such as posttraumatic stress disorder, PTSD), Asok et al. highlights the importance of sex differences and remote timescales in rodent models to improve the dialogue between human and animal studies. Factors that contribute to PTSD were also considered in the review by Bhattacharya et al. Recent research in both humans and animals suggests that experience of the parent may influence its offspring. Bhattacharya et al. review studies in both humans with PTSD and laboratory predator stress models of PTSD that suggest changes to DNA methylation may underlie the generational effects of trauma transmission. Both Asok et al., and Bhattacharya et al., argue that a deeper understanding of the mechanisms that promote stress-induced psychopathology will accelerate the development of effective therapeutics.

## Author Contributions

All authors listed have made a substantial, direct and intellectual contribution to the work, and approved it for publication.

### Conflict of Interest

The authors declare that the research was conducted in the absence of any commercial or financial relationships that could be construed as a potential conflict of interest.

